# Injectable Nanocomposite Biomaterial for 3D Printing of Personalized Matrices and Their Use in Bioreactors for Bioengineering Advanced Cell Culture Models

**DOI:** 10.1021/acsami.5c18437

**Published:** 2025-12-08

**Authors:** Elisabetta Campodoni, Andrea Mazzoleni, Margherita Montanari, Gaia Vicinelli, Valentina Possetti, Antonio Inforzato, Ivan Martin, Manuele G. Muraro, Monica Sandri

**Affiliations:** † Institute of Science, Technology and Sustainability for Ceramics (ISSMC)—National Research Council (CNR), Faenza, Ravenna 48018, Italy; ‡ Department of Biomedical Engineering, 27209 University of Basel, Basel CH-4001, Switzerland; § Department of Biomedical Sciences, 437807 Humanitas University, Pieve Emanuele 20072, Italy; ∥ IRCCS Humanitas Research Hospital, Rozzano, Milan 20089, Italy; ⊥ Tissue Engineering, Department of Biomedicine, 30262 University of Basel and University Hospital Basel, Basel CH-4001, Switzerland

**Keywords:** bone organoids, 3D printing, injectable biomaterials, perfusion bioreactor, bone tissue regeneration, hybrid hydroxyapatite, osteogenic differentiation

## Abstract

Printing technology is a leading strategy for creating customized 3D matrices for tissue engineering. Our study developed an injectable nanocomposite hydrogel (bHAGel) for high-fidelity 3D extrusion printing composed of gelatin (Gel) and magnesium-doped biomimetic hydroxyapatite (bHA) particles that mimics a bone extracellular matrix. bHA particles, synthesized through a bioinspired mineralization process, acted as multifunctional additives, modulating rheology for printability, ensuring homogeneous phase distribution, enabling excellent model fidelity, and providing osteoinductive cues. The optimized hydrogel formulation enables the fabrication of porous scaffolds with interconnected macro- and microporosity via extrusion-based printing and freeze-drying. This key feature promoted cell infiltration and nutrient diffusion during tissue engineering procedures. Biological validation involves tailoring 3D scaffolds to fit a perfusion bioreactor chamber supporting seamless handling, seeding, and long-term culturing without scaffold removal or repositioning. Dynamic in vitro experiments with donor-derived human bone marrow stromal cells assessed the constructs’ stability, ability to maintain geometry and perfusability, cytocompatibility and osteoconductivity, as well as robust osteogenic differentiation over 28 days. A more complex dynamic coculture model further demonstrated that the scaffold supports osteoclastogenesis under physiological, osteoblast-mediated conditions. Altogether, bHAGel scaffolds provided a customizable, bioactive platform suitable for engineering bone-mimetic organoids under dynamic conditions. Their modularity and biological relevance could be exploited in bone regeneration, disease modeling, and drug testing.

## Graphical abstract

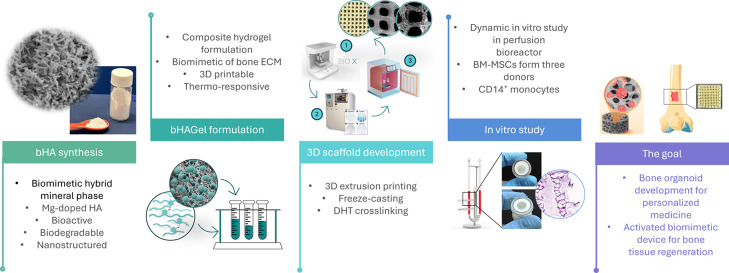

## 1 Introduction

Despite great breakthroughs in recent decades in illuminating the pathological mechanisms of bone-associated diseases and in developing targeted pharmacological therapies, it remains extremely challenging to find effective therapeutic solutions for most of them.^1–4^

Bone organoids, miniaturized in vitro structures recapitulating bone tissue, have gained attention for their advantages over conventional animal and two-dimensional (2D) cell models. However, existing in vitro systems are often limited to simple three-dimensional (3D) cultures or rely on immortalized cell lines such as mature bone cells and osteocytes, which are difficult to differentiate and maintain in culture. These models fail to capture key features of bone physiology, including structured mineralization, long-term remodeling, and multicellular dynamics.^5–8^ Therefore, to better understand pathophysiological mechanisms and advance targeted drug therapies, it is imperative to develop more physiologically relevant 3D models.^9^

Fluid dynamics studies and bioreactor design have contributed to the creation of biologically relevant stimuli that improve tissue functionality and organization. However, the effectiveness of these systems depends on the availability of compatible, mechanically stable, and biologically instructive scaffolds.^10–18^

Now it is clear that this path begins with the choice of the 3D biomatrices, suitable for use with dynamic culture techniques, and to emulate the native bone microenvironment with greater fidelity.^10^ Decellularized natural matrices offer excellent biological compatibility but suffer from batch variability and laborious processing.^19^

Synthetic biomaterials, while easier to manufacture, often lack biological specificity and are frequently incompatible with perfused dynamic cultures due to inadequate permeability or mechanical instability. Hydrogels and sponges, for instance, are rarely engineered with both the structural fidelity and the porosity required for controlled and sustained perfusion.^20–22^

In this scenario, 3D printing has emerged as a transformative strategy to generate architecturally controlled constructs with customized geometries. However, most printable hydrogel formulations lack multifunctionality and do not replicate the chemical and mechanical environment necessary to support bone-like tissue development.^23–25^

Biomimetic and nanocomposite hydrogels owing to their tunable aqueous chemistry and tissue-like physical behavior are promising candidates for bridging this gap.^20,26,27^ Yet, many hydrogel systems used for bioprinting require high cell numbers, are difficult to handle or store, and are incompatible with standardized workflows. Moreover, they seldom meet the combined criteria of printability, perfusability, and biological relevance.^28^

Therefore, it is evident that the significant challenges in implementing 3D cellular models and engineering bone grafts in research are the limited availability of tailor-made bioactive biomatrices that can support both fabrication precision and long-term reproducible functionality in perfused environments. Importantly, such scaffolds must mimic the in vivo microenvironment in terms of biocompatibility, osteoconductivity, and ideally osteoinductivity. They should feature a macroporous microstructure interconnected by micropores to facilitate cell growth, nutrient diffusion, and waste removal while maintaining mechanical stability during culture and being remodelable over time.

In this scenario, our research focused on the development of biomimetic, injectable nanocomposite hydrogels (bHAGels) composed of gelatin and hybrid magnesium-doped hydroxyapatite (bHA) particles, used to 3D print matrices that recapitulate the microenvironment of the bone niche as tools for the bioengineering of increasingly complex cellular models mimicking the pathophysiology of diseased tissues. bHA particles, synthesized via a bioinspired mineralization process, exhibit a crucial dual functionality: (i) they enhance the rheological behavior of the hydrogel to enable high-fidelity 3D printing and (ii) they introduce osteoinductive properties through their nanostructured, bioactive composition.^21,29–31^ The bHAGel formulation was optimized for extrusion-based printing, producing scaffolds with controlled macro-geometry. The integration of a freeze-drying step introduced a secondary microporous architecture, improving the internal surface area, fluid absorption, and compatibility with perfusion bioreactors. The resulting constructs demonstrated shape fidelity, dimensional reproducibility, and open porosity, which are essential for culture standardization and perfusion under a dynamic flow. To validate the functional suitability of the printed scaffolds, these were employed in a dynamic in vitro setting where human bone marrow-derived mesenchymal stromal cells (hBM-MSCs) were cultured for 28 days in a perfusion bioreactor. The scaffolds maintained structural stability, supported homogeneous cell colonization, and promoted osteogenic differentiation, as shown by ECM deposition and the gene expression of osteogenic markers.

Additionally, the printed constructs supported a second, more complex dynamic coculture involving both osteoblast- and osteoclast-lineage cells. Without the addition of supraphysiological stimulants, the bHAGel scaffold sustained osteoclastogenesis via osteoblast-mediated signaling, demonstrating the matrix’s capacity to support key elements of bone remodeling under physiological conditions.

## 2 Experimental Section and Methods

### 2.1 Development of the Injectable Hydrogel (bHAGel) and 3D-Printed Hybrid Scaffold

#### 2.1.1 Synthesis and Characterization of Biomimetic Hybrid Hydroxyapatite Particles (bHA)

For the bHA synthesis, a neutralization reaction was exploited. Three solutions were prepared: an acid solution containing 13.74 g of H_3_PO_4_ (Sigma-Aldrich, 85% pure) in 0.1 L of bidistilled water; a basic suspension containing 15.49 g of Ca­(OH)_2_ (Sigma-Aldrich, 95% pure) and 2.02 g of MgCl_2_·6H_2_O (Sigma-Aldrich) in 0.16 L of bidistilled water; and a polymeric solution containing 5 g of gelatin (Gel) powder (Italgelatin, Italy) in 0.1 L of bidistilled water prepared under stirring conditions at 45 °C. The H_3_PO_4_ solution was mixed with the gelatin solution until perfect blending and then slowly added to the basic suspension at 25 °C under constant and vigorous stirring. The mixture was stirred for 1 h, followed by a 2 h settling period at 25 °C. After centrifugation at 6000 rpm for 10 min at 25 °C, the pellet was collected, washed three times with bidistilled water by centrifugation, freeze-dried, sieved at 150 μm, and micronized at 3 μm. The morphology of bHA was evaluated with electron scanning microscopy (SEM, Carl Zeiss Sigma NTS Gmbh Öberkochen, Germany) after Pt coating (QT150T, Quorum Technologies Ltd., UK). Inductively coupled plasma–optical emission spectrometry (ICP–OES 5100, vertical dual view apparatus, Agilent Technologies, Santa Clara, CA, USA) and attenuated total reflection–Fourier transform infrared spectroscopy (ATR–FTIR, Thermo Fisher Scientific Inc., Waltham, USA) were used to determine the chemical characteristics and the stoichiometric composition of bHA. In detail, ICP allows for the quantification of the Mg^2+^, Ca^2+^, and PO_4_^3–^ ions, which constitute the inorganic mineral component; 10 mg of hybrid GelMgHA particles or 20 mg of scaffold was dissolved in 50 mL of a 2 wt % % HNO_3_ solution prior to the analysis. The crystallographic identity and crystallinity degree of bHA were evaluated with X-ray diffraction (XRD, Panalytical X’Pert PRO, Bruker, Germany). The analysis employed Cu Kα radiation (λ = 1.54178 Å) at 40 kV and 40 mA. Spectra were recorded in the 2θ range from 20° to 80 °C, with a step size (2θ) of 0.02° and a counting time of 0.5 s. Thermogravimetric analysis (TGA, STA 449/C Jupiter, Netzsch, Germany) was used to evaluate bHA weight losses under thermal treatment. The analysis was conducted in alumina crucibles, from room temperature to 1100 °C, at a heating rate of 10 °C/min under a nitrogen flow. The sample weighed approximately 10 mg.

#### 2.1.2 Formulation and Characterizations of the Injectable Composite Hydrogel (bHAGel)

bHAGel injectable formulations were prepared by varying bHA loading content, achieving 1:1, 0.5:1, and 0:1 w/w ratios of bHA with respect to Gel and maintaining constant a 5% w/V of Gel concentration (Table 1). Specifically, bHAGel_0 refers to a formulation without the mineral phase. Instead, to prepare 10 mL of composite hydrogel, 0.5 or 0.25 g of bHA was tip-sonicated with Na_3_C_6_H_5_O_7_ (Sigma-Aldrich) in 10 mL of bidistilled water for 10 min in an ice bath at 20% amplitude. Then, 0.5 g of Gel powder was added, and the mixture was kept at 45 °C under magnetic stirring for 1 h. In summary, bHAGel_0.5 has a bHA:Gel ratio of 0.5:1 and bHAGel_1 has a bHA:Gel ratio of 1:1.

**Table 1:** Composite Hydrogel Formulation

	gel (g)	bHA (g)	Na_3_C_6_H_5_O_7_ (g)	bidistilled H_2_O (mL)
bHAGel_1	0.5	0.5	0.112	10
bHAGel_0.5	0.5	0.25	0.056	10
bHAGel_0	0.5	0	0	10

The obtained hydrogel was loaded in 3 mL printer cartridges (CellInk, Göteborg, Sweden), and it was kept overnight at 4 °C and then thermally conditioned at 27 °C, the printing temperature, for 1 h, before printing.

The rheological properties were investigated by comparing the behavior of the three bHAGel formulations with different bHA loadings, measuring their viscosity against a shear rate ramp in controlled stress mode and against temperature, as well as their printability.

All of the measurements were performed with a rotational rheometer (C-VOR 120, Bohlin Instruments, UK). The shear ramp test was performed using a plate/plate PP20 (*Ø* = 20 mm) geometry, increasing the shear stress from 1 Pa up to 10,000 Pa with a sweep time of 720 s. A solvent trap was used to prevent water evaporation during the test. A temperature ramp test was performed using a plate/plate PP20 (*Ø* = 20 mm) geometry, increasing the temperature from 20 up to 35 °C, with a rate of 2 °C/min. Before the test was started, the samples were left to rest for 15 min to reduce any possible influence on the measurement because of the solution handling.

The printability of the biomaterial inks was assessed through three main tests: filament drop, filament spreading, and buildability (E (BIO X, CellInk, Göteborg, Sweden). The printability tests were executed with a *Ø*_in_ of 0.25 metal needle. For the filament drop test, after thermal conditioning, the filament extrusion profile of each biomaterial ink, extruded at the same pressure, was qualitatively evaluated. For the filament spreading test, 2 cm × 2 cm and 2 layers in height scaffolds were printed. High-resolution pictures of the printed structures were taken by digital optical microscopy (Hirox RH-2000, 3D Digital Microscope, Hirox Europe) and their model fidelity was determined using ImageJ software. Buildability of each ink was assessed by printing two different 3D structures: a 10 mm × 20 mm hollow cylinder and a 10 mm × 20 mm hollow cone.^32^

#### 2.1.3 3D Printing of Hybrid Scaffolds and Characterization

The 3D scaffolds were printed using a bioplotter (BIO X, CellInk, Göteborg, Sweden), with design and slicing software. The design consisted of a 2 cm × 2 cm square base and a 90° layer shift, resulting in a perpendicular mesh with 20% infill and a total height of 12 layers. Printing was performed with a *Ø*_in_ = 0.25 mm conical nozzle at an average pressure of 40 kPa and 5 mm/s speed. A thermocontrolled printhead (27 °C) and a cooled printbed (4 °C) were used. After printing, the obtained structures were freeze-dried (Freeze-drier, 5 Pascal Lio 3000 PLT, Rozzano, Milano, Italy) with a controlled freezing ramp of −50 °C/h until −40 °C, followed by a controlled heating of 5 °C/h from 40 °C to −5 °C and 3 °C/h until 20 °C, lasting approximately 3 days under a vacuum, *P* = 0.086 mbar. Finally, they were cross-linked via dehydrothermal treatment (DHT) at 160 °C for 72 h under vacuum 0.01 mbar (Vacuum Oven Heated Shelf 50 Lt, 5 Pascal Srl). Cylindrical scaffolds (8 mm in diameter) were punched out to fit the bioreactor chamber. Finally, the scaffolds were sterilized by 25 kGy of γ-ray irradiation.

The scaffold morphology was evaluated by environmental scanning electron microscopy (SEM TM Quanta 200, FEI, Thermo Fisher Scientific Inc.), set in High-Vacuum (*P* <10^–4^ Torr) mode. The samples were fixed on aluminum stubs using carbon tape, and they were coated with Au using a Polaron Sputter Coater E5100 (Polaron Equipment, Watford, Hertfordshire, United Kingdom). The macropore and micropore dimensions of the printed scaffolds were quantitatively evaluated from SEM micrographs using ImageJ (NIH, USA). To characterize the inclusion of bHA particles within the polymeric matrix and its interaction with it, XRD, ATR–FTIR, TGA, and ICP analyses were performed. The swelling behavior was assessed by soaking samples in 1× PBS solution with 0.1% w/v of NaN_3_ at 37 °C under shaking. Samples were weighed at different time points (0 h; 0.5 h; 3 h; 6 h; 24 h; 48 h) after 5 s rest on a nonabsorbent surface. The swelling ratio (*S*_r_) was calculated as

$$S_{r}=\frac{W_{s}-W}{W}$$

where *W*_s_ is the swollen sample weight and *W* the dry sample weight before soaking.

Degradation analysis was performed in the incubator exposing the samples to a 1× PBS solution with 0.1% w/v NaN_3_ at 37 °C at different time points (3, 7, 14, 21, 28 days), washing them three times with bidistilled water before freeze-drying and weighing them.

The degradation ratio (*D*_%_) was calculated as

$$D_{\%}=\frac{W-W_{d}}{W_{d}}\times 100$$

where *W* is the dried sample initial weight and *W*_d_ is the degraded sample weight at a specific time point.

Dynamic mechanical analysis (DMA) was performed with wet samples submersed in PBS and at 37 °C to better replicate physiological conditions. Compression test was performed at 37 °C by using a Q800 DMA (TA Instruments, USA). The samples, with dimensions of 8 × 3 mm (*Ø* × *h*), were incubated in PBS overnight prior to testing and were preloaded to 0.01 N to ensure full contact between the scaffold surfaces and the compression plates. Then, compression testing was performed compressed in force control using a force ramp rate of 0.5 N/min to the upper force limit of 5 N. The compressive moduli were calculated as the slope of the initial linear part of the stress–strain curve up to 15% strain.^33^

### 2.2 3D Scaffold Dynamic Culturing and Biological Evaluation

#### 2.2.1 Cell Source and Expansion

Bone marrow (BM) aspirates (Table 2) were obtained during routine orthopedic surgical procedures involving exposure of the iliac crest, after informed consent from the patient and following protocol approval by the local ethical committee (Ethical Komission Beider Basel #78/07). BM mesenchymal stromal cells (BM-MSCs), selected based on adhesion and proliferation on the plastic substrate as previously described, were used after two passages of expansion.^32^

**Table 2:** Bone Marrow Samples Used for In Vitro Assessments

BM sample ID	sex	age (years)
BM187	male	43
BM231	male	17
BM272	female	47

Freshly isolated nucleated cells were plated at a density of 1*10^5^ cells/cm^2^. Cell expansion was carried out in complete medium (CM), which consisted of α-modified Eagle’s medium (α-MEM), 10% fetal bovine serum, 1× GlutMAX, 100 mM HEPES buffer solution, 1 mM sodium pyruvate, 100 U/ml penicillin, 100 mg/mL streptomycin (Thermofisher Gibco), and supplemented with 5 ng/mL fibroblast growth factor-2 (FGF-2; R&D Systems). The medium was changed twice a week. At confluence, human bone marrow mesenchymal cells (hBM-MSCs) were replated for expansion (seeding density of 3–5*10^3^ cells/cm^2^). Upon confluence, cells at passage 2 or 3 were enzymatically retrieved and counted for use in the 3D culture experiments.

#### 2.2.2 3D Culture System

Dynamic 3D cultures were established using the perfusion-based U-CUP bioreactor system (CELLEC Biotek AG), previously validated for uniform cell seeding and long-term culture within 3D scaffolds.^32^ A schematic overview of the experimental design and workflow, including scaffold installation, cell seeding, perfusion culture phases, and end point analyses, is provided in Figure 1.

**Figure 1:**
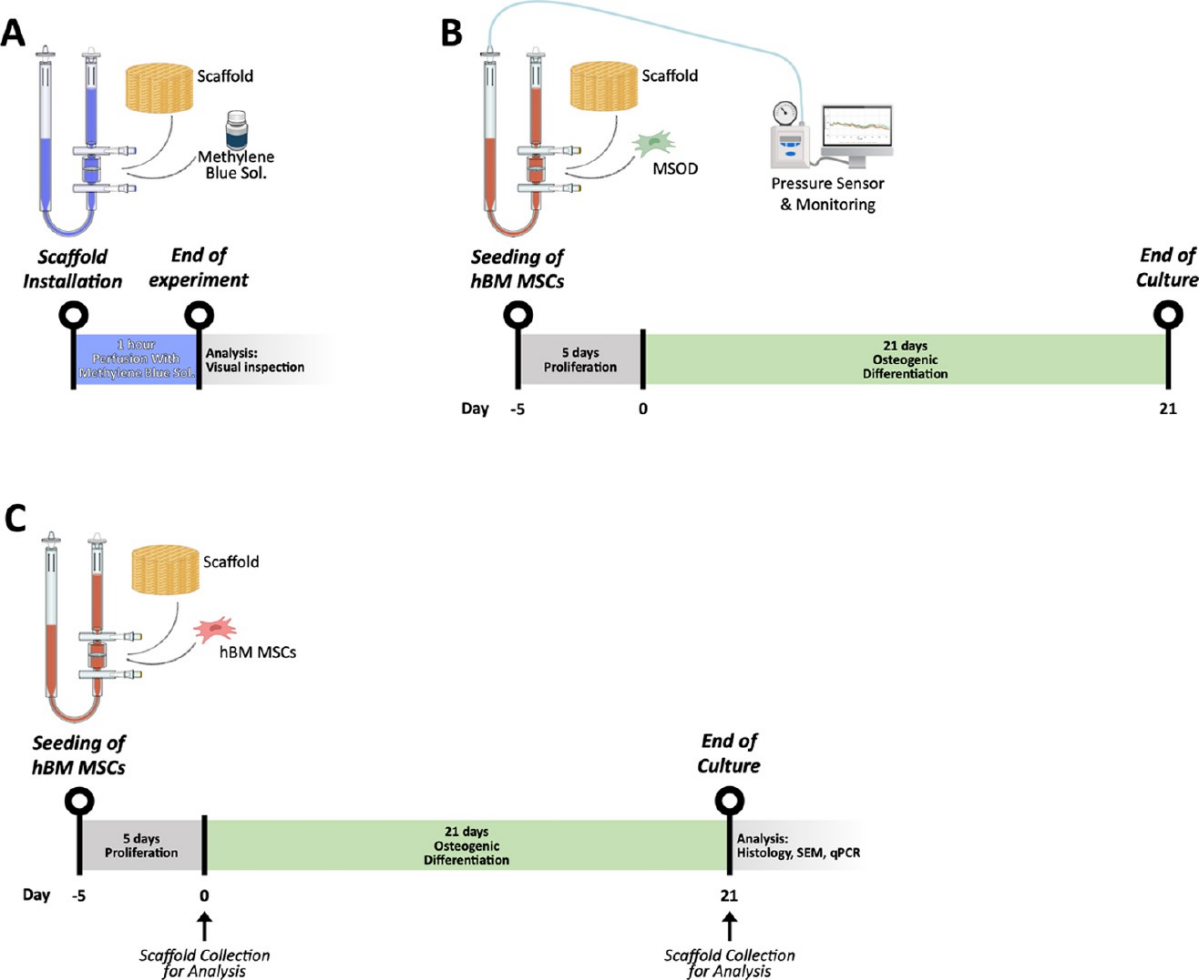
Experimental design and workflow of scaffold installation and perfusion-based culture. (A) Scaffold installation and 1 h perfusion with methylene blue solution to verify uniform fluid distribution. (B) Dynamic culture of MSOD-seeded scaffolds under perfusion: 5 days proliferation followed by 21 days osteogenic differentiation with continuous pressure monitoring. (C) Dynamic culture of hBM-MSC-seeded scaffolds under the same timeline. Day 0 marks the start of differentiation, and scaffold samples were collected for histological, SEM, and qPCR analyses.

In the first set of experiments, 1 × 10^6^ human BM-MSCs were suspended in 8 mL of CM supplemented with 5 ng/mL FGF2, 10 nM dexamethasone, and 0.1 mM l-ascorbic acid-2-phosphate (DAF medium), a preconditioning for attachment and proliferation.^34–36^ The suspension was perfused through the bHAGel scaffold (*Ø* 8 mm × 4 mm) at a superficial velocity of 1 mm/s (corresponding to 3.00 mL/min). Following 5 days of dynamic culture, the medium was replaced with osteoinductive medium (OM), consisting of CM supplemented with 10 nM dexamethasone, 0.1 mM l-ascorbic acid-2-phosphate, and 10 mM β-glycerophosphate, and the culture was maintained for an additional 3 additional weeks.

To investigate osteoclast differentiation, 2 × 10^6^ freshly isolated CD14^+^ monocytes from buffy coats obtained from healthy donor’s peripheral blood were seeded on osteo-induced MSC scaffolds and cocultured for 10 days under perfusion in CM supplemented with 10 nM vitamin D3 and 1 μM Prostaglandin E2 (PGE2). The medium was renewed every 2–3 days.

In parallel, static cocultures were performed to compare scaffold performance and cellular interactions. Briefly, 60 μL of a suspension containing 1 × 10^5^ MSCs was pipetted directly onto the bHAGel scaffolds and incubated for 40 min at 37 °C in low-attachment 12-well plates (Sarstedt, #4021721) to allow cell attachment. Scaffolds were then submerged in DAF medium and maintained for 5 days. Subsequently, the medium was switched to OM and the culture continued for 3 weeks. CD14^+^ cells (1 × 10^6^) were then seeded onto the constructs and allowed to attach for 40 min at 37 °C. Cocultures were maintained for 10 additional days in CM supplemented with 25 ng/mL M-CSF and either 10 nM vitamin D3 (osteotropic condition) or 50 ng/mL RANKL (osteoclastogenic condition). Experiments were conducted with BM-MSCs from three donors (BM187, BM231, BM272). Dynamic osteogenic cultures included *n* = 2 scaffolds per donor at day 0 and *n* = 3 at day 21; acellular scaffolds (*n* = 3) served as material controls. Each scaffold was cultured in an independent bioreactor unit. Osteoblast–osteoclast cocultures (donor BM272) were performed with *n* = 2–3 independent bioreactor replicates.

#### 2.2.3 Scanning Electron Microscopy

Engineered tissue constructs were fixed overnight at 4 °C in 0.1 M sodium cacodylate buffer containing 2% glutaraldehyde and then dehydrated through a graded ethanol series (30% to 100%, 15 min each). Samples were dried using critical point drying with liquid CO_2_ (Autosamdri-815, Tousimis), mounted on aluminum stubs, and sputter-coated with 20 nm gold (LEICA EM ACE600). SEM imaging was performed on a ZEISS Gemini 2 microscope at 5 kV and 200 pA, with magnifications from 500 to 5000×. Surface microstructure was qualitatively evaluated on longitudinal and transverse sections.

#### 2.2.4 Histological and Immunohistochemical Staining

Paraffin-embedded scaffold samples were sectioned at 7 μm thickness using a microtome (Microm HM 355S, Thermo Scientific). Sections were mounted on Superfrost Plus glass slides (Thermo Scientific), deparaffinized with Ultraclear (Bio-Optica), and rehydrated through a graded ethanol series (100%, 96%, 70%, and 50%; two washes per step, 10 min each) into distilled water.

Hematoxylin and eosin (H&E) staining was performed by immersing sections in Harris hematoxylin for 7 min, rinsing under running tap water, and counterstaining with eosin for 2 min. Slides were subsequently dehydrated, cleared, and mounted with coverslips.

TRAP staining was performed on separate section sets to detect osteoclast activity. Sections were incubated in 0.2 M acetate buffer containing TRAP5b staining solution (1 mg/mL Naphthol AS-MX Phosphate Disodium Salt and 1 mg/mL Fast Red Violet LB Salt; Sigma-Aldrich) at 37 °C for 1–4 h. After washing in distilled water, sections were counterstained with Mayer’s hematoxylin for 1 min, rinsed in running water, air-dried, mounted with Pertex (Histolab), and imaged using a fluorescence microscope (Nikon TI2).

Immunohistochemistry (IHC) was conducted using a Ventana Discovery Ultra platform (Roche Diagnostics) with primary antibodies against bone sialoprotein (BSP; Abcam ab52128, 1:100) and Osteopontin (OPN; Proteintech 22952-1, 1:100). Antigen retrieval and detection followed the manufacturer’s automated protocol. Counterstaining was performed with hematoxylin.

#### 2.2.5 Immunofluorescence

Paraffin-embedded scaffold sections were deparaffinized in xylene and rehydrated through a graded ethanol series (100%, 96%, 70%, and 50%; two washes per step, 10 min each). Antigen retrieval was performed by incubating slides at 95 °C for 30 min in citrate buffer (pH 6.00, ProTaqs, no. 400300692), followed by cooling at RT for 20 min. To permeabilize the tissue, sections were washed twice for 10 min with 1% goat serum (GS) in PBS containing 0.4% Triton X-100 (PBS-T). Nonspecific antibody binding was blocked with 5% GS in PBS-T (blocking solution, BS) for 30 min at RT.

Sections were incubated overnight at 4 °C with a rabbit antihuman PTX3 polyclonal antibody (in-house purified) diluted 1:200 in PBS-T containing 1% GS. The next day, sections were washed twice in the same buffer (10 min each), then incubated for 1 h at RT with an Alexa Flour 647-conjugated goat antirabbit IgG (H + L) polyclonal secondary antibody (Invitrogen, #A21245) diluted in PBS-T containing 1% GS. After two additional 10 min washes, nuclear counterstaining was performed using DAPI (1:1000 in PBS; BD Biosciences, #564907) for 5 min. Slides were rinsed in PBS, mounted, and imaged using a fluorescence microscope (Nikon TI2).

For the whole-mount immunostaining, selected 3D scaffold constructs were harvested at the end of the coculture period, cut in two halves, and fixed overnight in 4% paraformaldehyde at 4 °C. After washing in PBS, scaffolds were permeabilized with PBS-T for 10 min and blocked for 1 h at 4 °C in BS. Primary antibodies, rabbit anti-osteocalcin polyclonal (Abcam, #ab93876) or rabbit recombinant anti-TRAP monoclonal (Abcam, #ab240970), were diluted 1:200 in BS and incubated overnight at 4 °C. Samples were washed twice with PBS, then incubated for 1–4 h at RT with an Alexa Flour 647-conjugated goat antirabbit polyclonal secondary antibody (Abcam, #a21247) diluted 1:1000 in BS. After two PBS washes, nuclei were counterstained with DAPI (1:1000 dilution in PBS, 10 min, RT). Scaffolds were loaded onto multiwell imaging slides (ibidi, #80821) and imaged using a Nikon AXR confocal microscope.

#### 2.2.6 Immunoassays

Quantification of PTX3 and OPG in OB/OC coculture supernatants was performed using commercial ELISA kits, following the manufacturers’ protocols (PTX3: Hycult Biotech; OPG: R&D Systems). Absorbance was measured using a VERSAmax Tunable Microplate Reader, and data were analyzed with SoftMax Pro 5.3 software (Molecular Devices, LLC.).

The concentrations of human MMP9 and BMP9 in the OB/OC coculture were assessed using the ELLA Automated Immunoassay System (Bio-Techne), in accordance with the manufacturer’s instructions. The reported limits of detection (LODs) were 0.156 ng/mL for MMP9 and 0.086 pg/mL for BMP9.

#### 2.2.7 RNA Extraction and Quantitative Real-Time PCR

Total RNA was extracted using the Trizol reagent (Invitrogen, Thermo Fisher) following mechanical homogenization with stainless steel beads in a TissueLyser II system (Qiagen). RNA concentration and purity were assessed via NanoDrop One spectrophotometry (Thermo Scientific). One microgram of RNA was reverse-transcribed using M-MLV reverse transcriptase (Invitrogen) according to the manufacturer’s protocol. Quantitative real-time PCR (qRT-PCR) was performed using TaqMan Gene Expression Assays on a ViiA 7 Real-Time PCR System (Applied Biosystems). Target genes included Runx2, Osteocalcin (Bglap/Ocn), Bone Sialoprotein (Ibsp), Collagen type I (Col1a1), Bone Morphogenic Protein 2 (Bmp2), Tartrate Resistant Acid Phosphatase (Trap), Cathepsin K (Ctsk), Osteopontin (OPN), and Pentraxin-3 (Ptx3). GAPDH was used as the endogenous control, and the relative gene expression was calculated using the 2^–Δ*Ct*^ method.

## 3 Results and Discussion

### 3.1 Development of the 3D-Printed Hybrid Scaffold (bHAGel)

This work develops a hybrid scaffold composed of gelatin (Gel) as the primary polymeric matrix and biomimetic hybrid particles composed of nanostructured magnesium-doped hydroxyapatite (Mg-doped HA, referred to as bHA) that is grown on gelatin molecules. The scaffold was fabricated using a combination of 3D printing and freeze-drying techniques to create bimodal porosity, featuring both macro- and micropores, designed to enhance cell adhesion and proliferation in a dynamic in vitro cell culture system (Figure 2). The scaffold was then cross-linked through dehydrothermal treatment (DHT) to improve its resistance to degradation and enable its use for long-term cultures.

**Figure 2:**
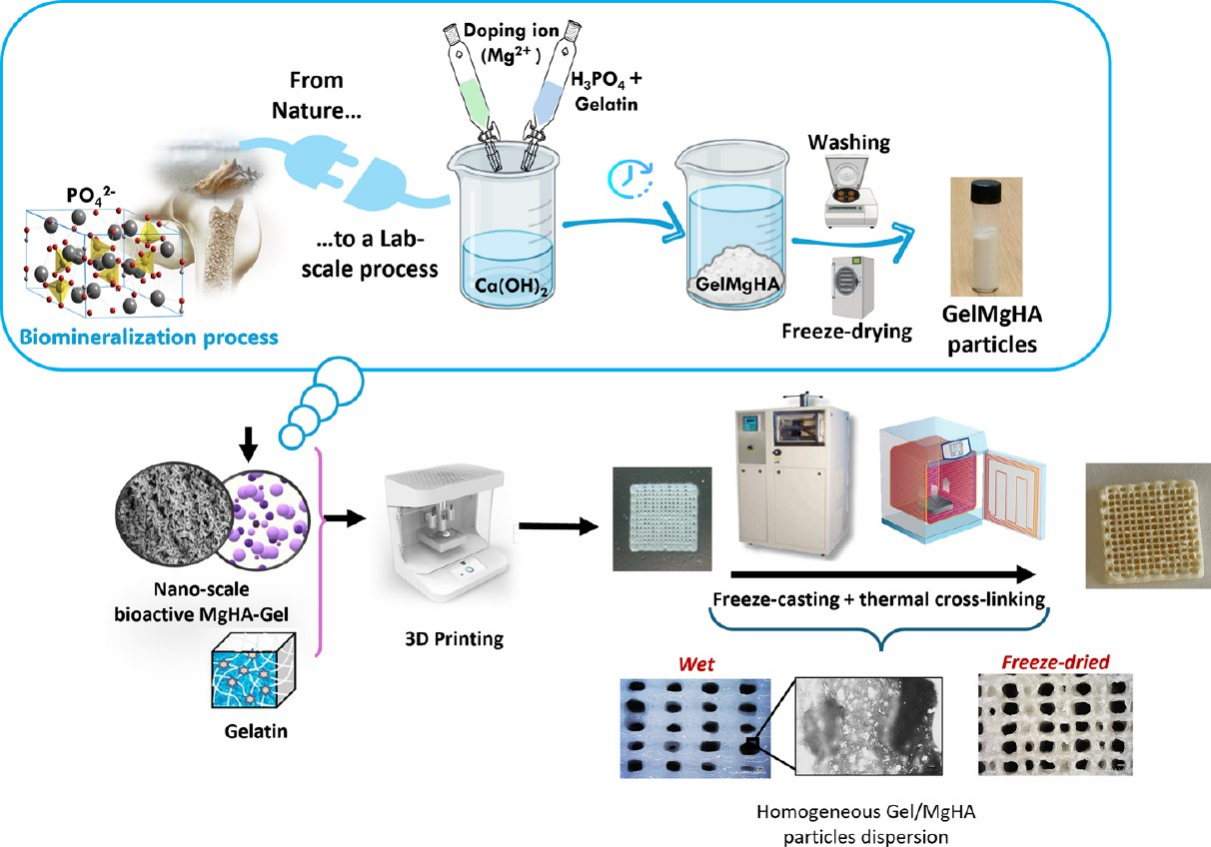
Schematic illustration of the step-by-step process for the synthesis of the injectable nanocomposite hydrogel (bHAGel) and of the 3D-printed scaffold developed and investigated in this study.

#### 3.1.1 Synthesis and Characterization of Hybrid Hydroxyapatite (bHA)

The naturally occurring biomineralization process was translated into an in-lab process to create a bioinspired and highly biomimetic hydroxyapatite (bHA). In detail, Mg^2+^-doped HA was nucleated and grown directly onto the Gel macromolecules. The in-lab biomineralization process was achieved by performing a neutralization reaction in an intentionally uncontrolled environment (air) at a low temperature (Troom), physiological pH conditions, in the presence of Mg^2+^ as doping ion and Gel as the organic template.

An open-air reaction environment favored spontaneous doping with CO_3_^2–^ ions derived from CO_2_, thus reproducing a physiological aspect of natural bone deposition. Mg^2+^ doping, typical of newly formed bone, promoted acceleration of HA nucleation kinetics, consequently reducing its crystallinity. The acidified gel provided nucleation centers for biomineralization, working in conjunction with the low synthesis temperature and doping ions to hinder crystallization, thereby enhancing the final biomimicry of the obtained bHA.^37^

Conceptually inspired by the in-lab collagen biomineralization process reported by Tampieri et al. in 2008,^38^ this method instead utilizes Gel as a template, enabling the formation of biomineralized microparticles rather than a three-dimensional structure. These hybrid microparticles are well suited as mineral additives in ink formulations for 3D printing and bioprinting. The presence of Gel combined with low temperature allowed for the growth of nanoparticles of hydroxyapatite with reduced crystallinity. The hybrid microparticles appear as microsized flakes, and it is possible to observe the presence of Gel inside the particle (Figure S1), whereas the nanostructured surface featured needle-like hydroxyapatite nanoparticles (Figure 3A).

**Figure 3:**
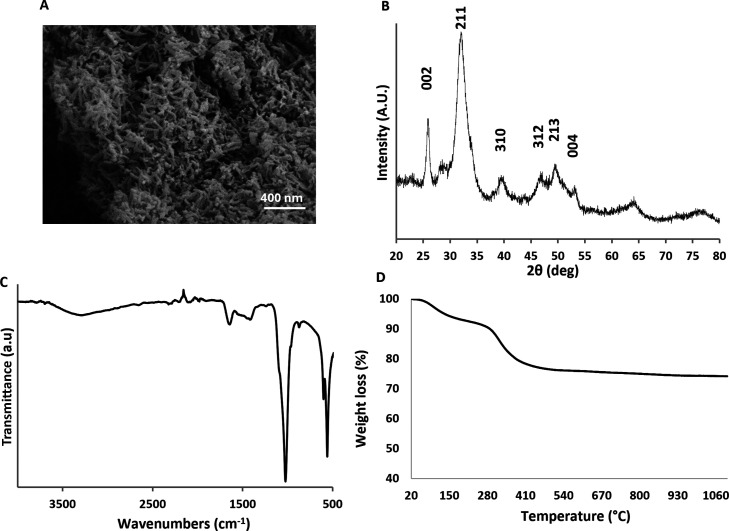
Morphological and physicochemical characterization of bHA particles. (A) SEM of bHA nanoparticles; (B) XRD spectrum of bHA; (C) ATR–FTIR spectrum of bHA; (D) TGA profile of bHA.

Crystallographic analysis (Figure 3B) revealed the presence of characteristic signals of hydroxyapatite (according to PDF card #09-0432), which appeared rather broad. This indicates the formation of poorly crystalline mineral phases, a consequence of the biomineralization process carried out at low temperature, that determines the crystal growth in interaction with gelatin molecules. This interaction hinders the formation of a perfectly ordered crystal structure, promoting the formation of small crystallites.^39^ Through ATR–FTIR analysis (Figure 3C), both inorganic and organic components were detected. The organic component, attributable to gelatin (Gel), was identified by the presence of characteristic amide I and II bands at around 1650 cm^–1^. However, due to the low organic/inorganic ratio (20/80) of bHA, the Gel-related peaks appeared poorly defined, though still clearly detectable.^40^ On the other hand, the prevailing presence of the inorganic component, made of hydroxyapatite, was recognizable by the well-defined phosphate signals within the range of 450–1200 cm^–1^. The thermogravimetric analysis confirmed the inorganic/organic ratio of 20/80 (Figure 3D).

#### 3.1.2 Formulation and Characterizations of the Biocomposite Injectable Hydrogel (bHAGel)

The rheological assessment performed on the bHA ink indicated that this has a shear thinning behavior with a viscosity of 33.9 kPa at 27 °C, the printing temperature, making it suitable for an effective printing process.

The rheological analysis performed at 27 °C (Figure 4A) confirmed the shear-thinning behavior of all tested formulations, a critical property for extrusion-based printing. Increasing the content of biomimetic hydroxyapatite (bHA) did not alter the overall shear-thinning profile, indicating that all formulations maintained excellent flow characteristics under applied stress. This behavior ensures consistent extrusion performance and reduces the risk of nozzle clogging—a key requirement for precision printing.

**Figure 4:**
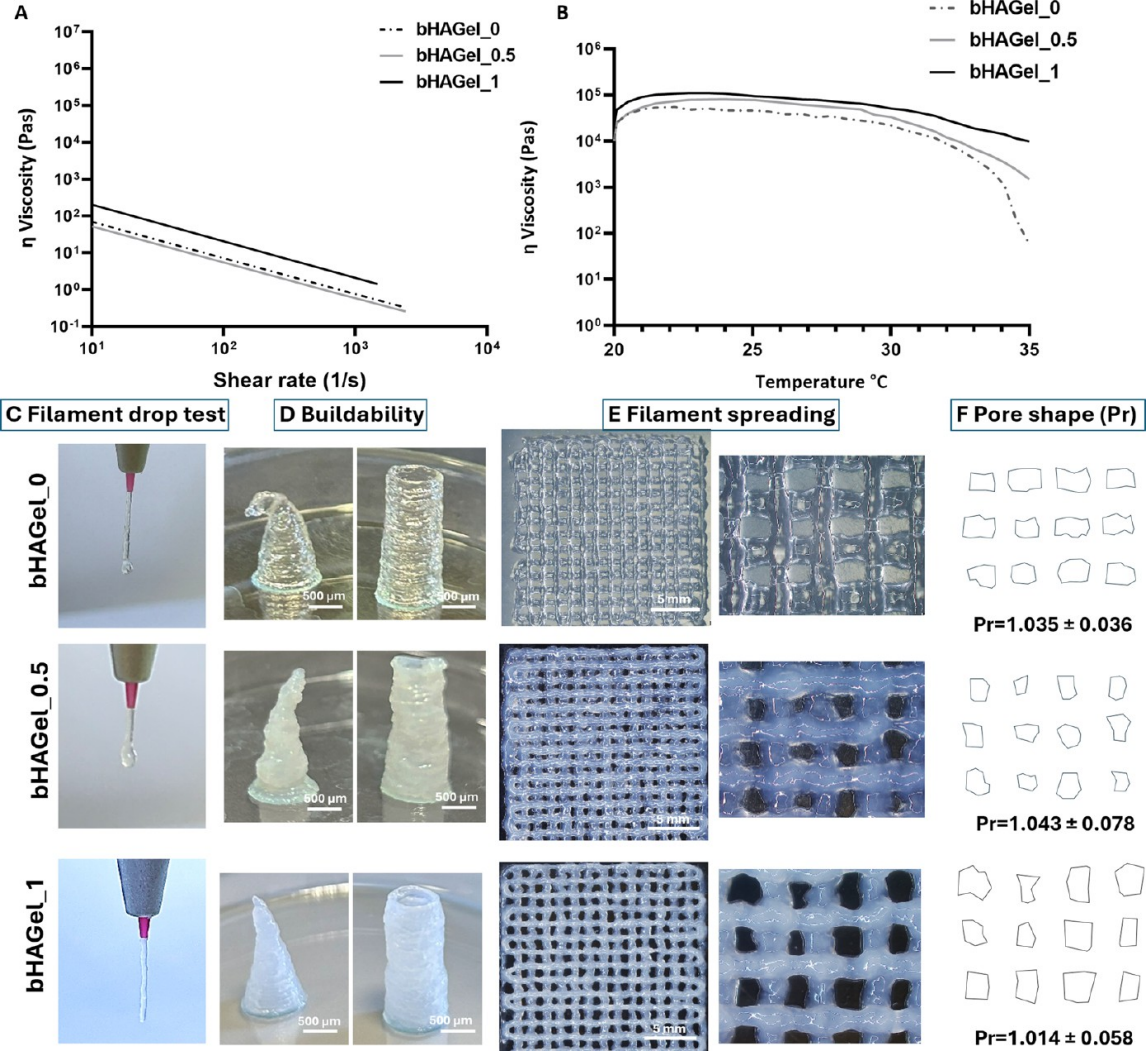
Rheological characterizations of biocomposite inks with different bHA content from 0% to 50% (bHAGel_0, bHAGel_0.5, bHAGel_1) as detailed in the Experimental Section and Methods: (A) shear rate dependency; (B) temperature dependency; (C) filament drop test; (D) buildability; (E) filament spreading, scale bar 5 mm, and (F) pore shape image and illustration with printability factor (Pr) values calculated (*n* = 30).

Moreover, bHA particles have previously been shown to enhance the rheology of gelatin-based systems, acting as multifunctional additives that not only provide osteogenic cues but also improve structural fidelity during printing.^41^ The viscosity–temperature profile (Figure 4B) further highlights the thermoresponsive nature of the Gel matrix, with a general decrease in viscosity observed as temperature increases. Interestingly, a modest initial increase in viscosity was detected at low-to-moderate temperatures before the expected thermal thinning. This behavior can be attributed to temperature-activated associative interactions between polymer chains and bHA nanoparticles, which transiently strengthen the network structure before the onset of thermal disruption at higher temperatures. This trend reflects the increased molecular mobility of Gel chains at elevated temperatures. Notably, the bHAGel_1 formulation exhibited a more stable viscosity profile across the temperature range, due to the higher mineral phase content. The abundant bHA appears to hinder Gel chain mobility, thereby moderating the viscosity drop and contributing to better temperature stability, a crucial feature for maintaining consistent printability across varying conditions.

The printability of the developed biomaterial inks was evaluated in terms of extrudability, buildability, and model fidelity, as summarized in Figure 4C–F. The filament drop test demonstrated a consistent and continuous flow across all formulations, with filament diameters matching the nozzle size. Notably, the bHAGel_1 sample showed minor swelling and curling, suggesting that the inclusion of 50% bHA provides adequate pregelation and an optimal printing temperature for stable extrusion. This test, which reflects the initial gelation state of the ink, is critical for achieving well-defined 3D structures with controlled porosity. Indeed, the cylindrical and conical constructs printed (Figure 4D) revealed that increasing the mineral content improved the buildability of the structures, preventing collapse or deformation. This suggests that the addition of bHA nanoparticles enhances the ink’s flow behavior by shifting its viscoelastic response from elastic to plastic, without compromising the rapid temperature-triggered gelation typical of thermosensitive hydrogels. Filament spreading analysis (Figure 4E) was used to assess structural fidelity semiquantitatively. The Pr factor (pore shape factor) revealed that all tested inks generated pores with shapes close to the ideal square geometry (Pr ≈ 1), falling within the defined printability range of 0.9 ≤ Pr ≤ 1.1.^42^ This indicates that, despite differences in buildability, all inks are printable and capable of forming regular porous patterns. Interestingly, the slightly higher Pr values (>1) observed in some formulations suggest a more gelled state at the time of extrusion, which—while potentially reducing flow—enhanced shape retention and structural accuracy postdeposition. As emphasized in previous studies,^43^ printability is inherently multifactorial, and no single parameter can fully capture the performance of a biomaterial ink. Considering these favorable rheological characteristics, the reliable printability, and the compositional and structural resemblance to the native bone tissue, the bHAGel_1 formulation was selected as the bioink of interest for this study. For simplicity, it will hereafter be referred to as bHAGel.

#### 3.1.3 3D Printing of Composite Matrices and Characterization

Gel was used both as a biomineralization template for bHA and an organic matrix to develop composite biomaterial ink.^44^

To this end, sodium citrate was employed to improve bHA colloidal stability by promoting its electrostatic interaction with the Gel matrix, preventing sedimentation and a consequent uneven distribution of the biomineralized phase within the layers during printing.^45,46^ This specifically considered the high loading of bHA (1:1 with respect to the organic matrix).

The development of dual-porosity scaffolds, achieved by combining 3D printing with freeze-drying, represents a crucial strategy for engineering constructs intended for bone regeneration in perfusion bioreactors. While 3D printing enables the fabrication of well-defined macroporous architectures with controlled filament orientation and interconnectivity, it alone does not generate the microporous texture necessary to fully mimic the native extracellular matrix of bone.^47^ To investigate the influence of drying methods on porosity, we compared freshly printed filaments, air-dried filaments, and freeze-dried filaments (Figure 5). In both freshly printed and air-dried samples, the observed porosity was limited to the macropores defined by the 3D printing design. In contrast, freeze-dried samples exhibited a secondary level of porosity, characterized by an interconnected microporous network within the filaments themselves, resulting from ice sublimation during the lyophilization process.

**Figure 5:**
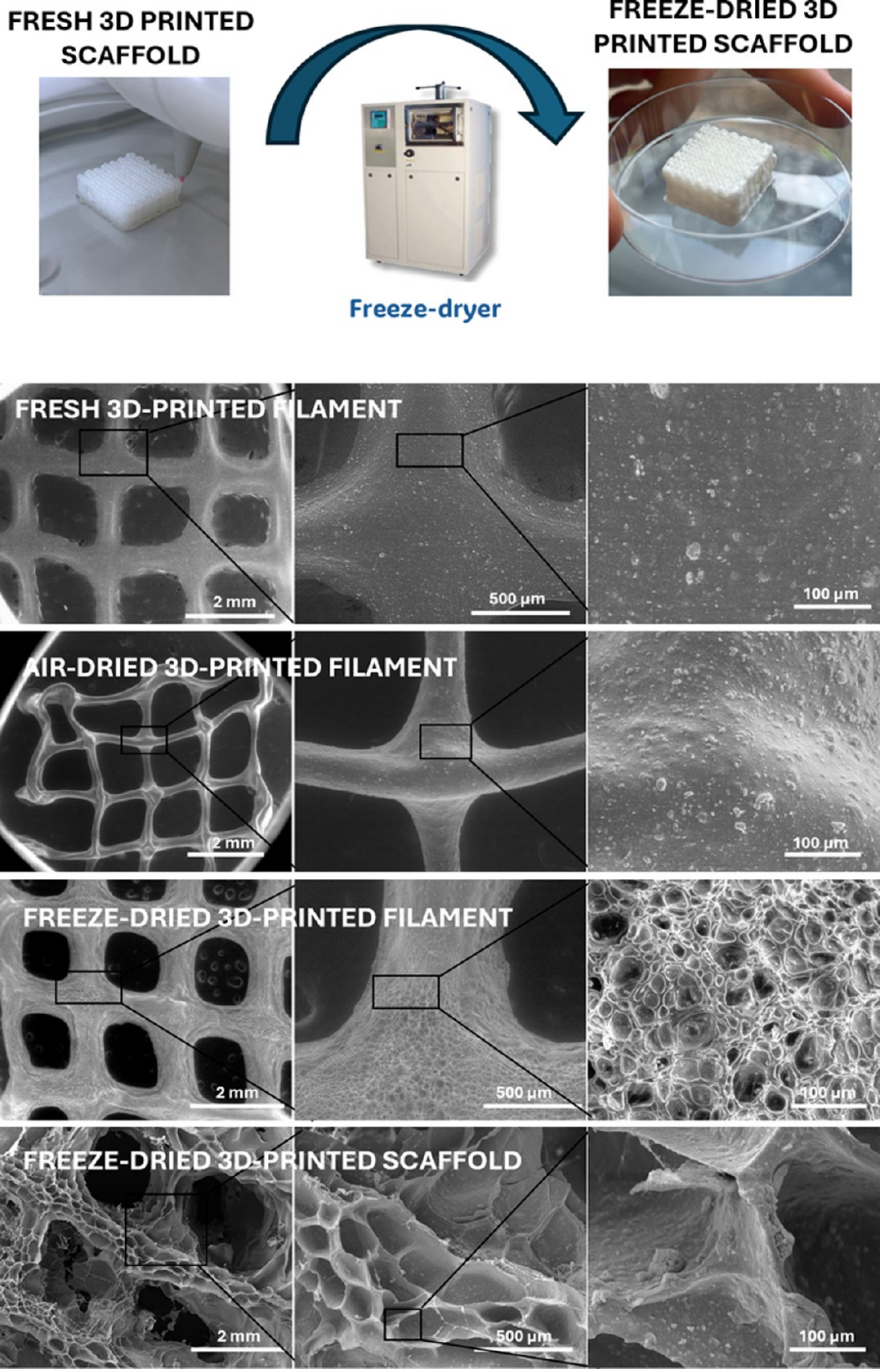
Morphological evaluation of different filaments of bHAGel. Fresh 3D-printed filament; air-dried 3D-printed filament; freeze-dried 3D-printed filament; freeze-dried 3D-printed scaffold.

Quantitative image analysis (using ImageJ thresholding and contour detection calibrated on the scale bars) revealed that the macropores between printed filaments exhibited an average diameter of approximately 800–900 μm, while the internal micropores within each filament ranged between 80 and 100 μm. Such hierarchical organization closely matches the dual-scale porosity typically considered optimal for bone tissue engineering, where macropores (>300 μm) support vascularization and tissue ingrowth, and micropores (10–150 μm) enhance cell attachment, nutrient diffusion, and matrix deposition.^47,48^ This microporosity enhances the internal surface area, facilitates the diffusion of nutrients and oxygen, and improves cell infiltration and matrix deposition under dynamic culture conditions. Thus, the synergy between structural macroporosity from 3D printing and textural microporosity from freeze-drying is crucial for designing biofunctional scaffolds that are compatible with perfusion bioreactors and can support effective bone tissue regeneration.^49^

A physicochemical characterization of the bHAGel composite was performed to confirm that the properties of bHA were preserved during the 3D printing process. XRD analysis (Figure 6A) demonstrated that the bHA particles retained their low crystallinity; the diffractogram is broader for the significant presence of gelatin. Thermogravimetric analysis (Figure 6B) revealed a distinct profile due to the addition of the Gel phase, confirming a bHA loading of 50% w/w. This composition resulted in an overall organic-to-inorganic ratio of 58:42 in the final injectable formulation. Interestingly, the ATR–FTIR profile showed, compared to that of bHA, increased peaks corresponding to the primary and secondary amides around 1650 cm^–1^, typical of Gel, and consistent with the increased amount of Gel in the formulated composite biomaterial ink (Figure 6C).

**Figure 6:**
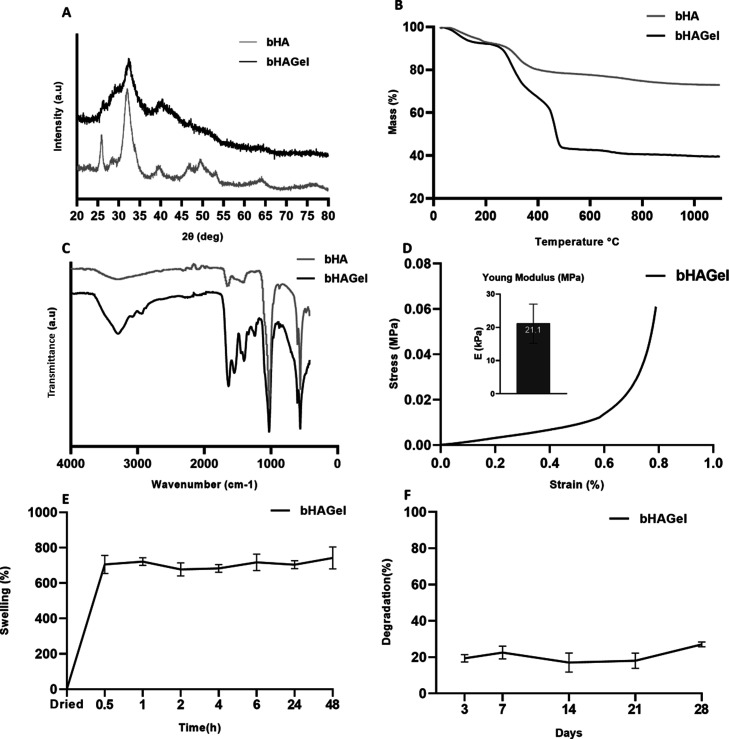
3D-printed scaffold characterizations. (A) XRD spectrum; (B) TGA profile; (C) ATR–FTIR spectrum of the bHAGel scaffold (black) in comparison with bHA mineral particles (lighht gray); (D) stress–strain test to evaluate the Young modulus (inset) of the bHAGel scaffold; (E) swelling performance of the bHAGel scaffold; (F) degradation rate of the bHAGel scaffold.

Since the last aim of this construct is to be fitted in a bioreactor, testing its mechanical properties in bioreactor-like conditions becomes crucial to better understand and predict its performance in long-term cultures. bHAGel was tested overnight preconditioned in PBS at 37 °C and then tested in uniaxial submersion-compression mode at the same conditions (Figure 6D). The Young Modulus (*E*), determined as the angular coefficient of the stress–strain response from 5 to 15% strain, was found to be 21.1 kPa. This value is within the typical range reported for soft hydrogels used in tissue engineering applications and is particularly well-suited to mimicking the mechanical microenvironment of early bone marrow or trabecular bone niches. While not as stiff as the native cortical bone, which is in the GPa range, this level of stiffness is adequate for perfusion bioreactor culture and supports cell proliferation, migration, and extracellular matrix deposition under dynamic conditions.

Since the dried scaffold is intended for use under wet conditions, its swelling behavior was evaluated after incubation in medium at 37 °C for different time points, as well as its degradation profile under static conditions up to 28 days (Figure 6E,F). bHAGel exhibited a high swelling capacity reaching approximately 700% within the first hour. This confirms the successful generation of bimodal porosity, which facilitates efficient cell infiltration and nutrient diffusion. The pronounced swelling can be ascribed to the hydrophilic nature of the polymeric matrix and the presence of interconnected pores that allow rapid water uptake through capillary forces.^50–52^ Importantly, the high swelling did not compromise scaffold stability, as demonstrated by a limited degradation of about 20% after 28 days under cell culture-like conditions. The relatively slow degradation rate is likely due to the stable cross-linked network that resists hydrolytic cleavage, ensuring the maintenance of structural integrity over prolonged incubation.^53–55^ Overall, these features support the suitability of bHAGel for long-term in vitro applications, where a balance between water absorption and mechanical stability is crucial.

### 3.2 3D Scaffold Dynamic Culturing and Biological Evaluation

Dynamic perfusion was employed to facilitate homogeneous cell seeding and to improve scaffold colonization by enhancing fluid-mediated cell transport throughout the construct.^56,57^ While perfusion systems can provide mechanical stimulation known to influence osteogenic pathways via shear-induced mechanotransduction, this study focused on evaluating matrix-driven effects and did not isolate flow-specific contributions. The compatibility of the bHAGel scaffold with perfusion-based culture is supported by its tailored structural and rheological properties: the hydrogel formulation enabled reproducible 3D printing of interconnected macroporous architectures, while the freeze-drying process introduced microporosity that enhanced the permeability. In combination with the mechanical stability imparted by the bHA component, these features ensured scaffold integrity and responsiveness to dynamic flow throughout the 28-day of culturing (Figure 7).

**Figure 7:**
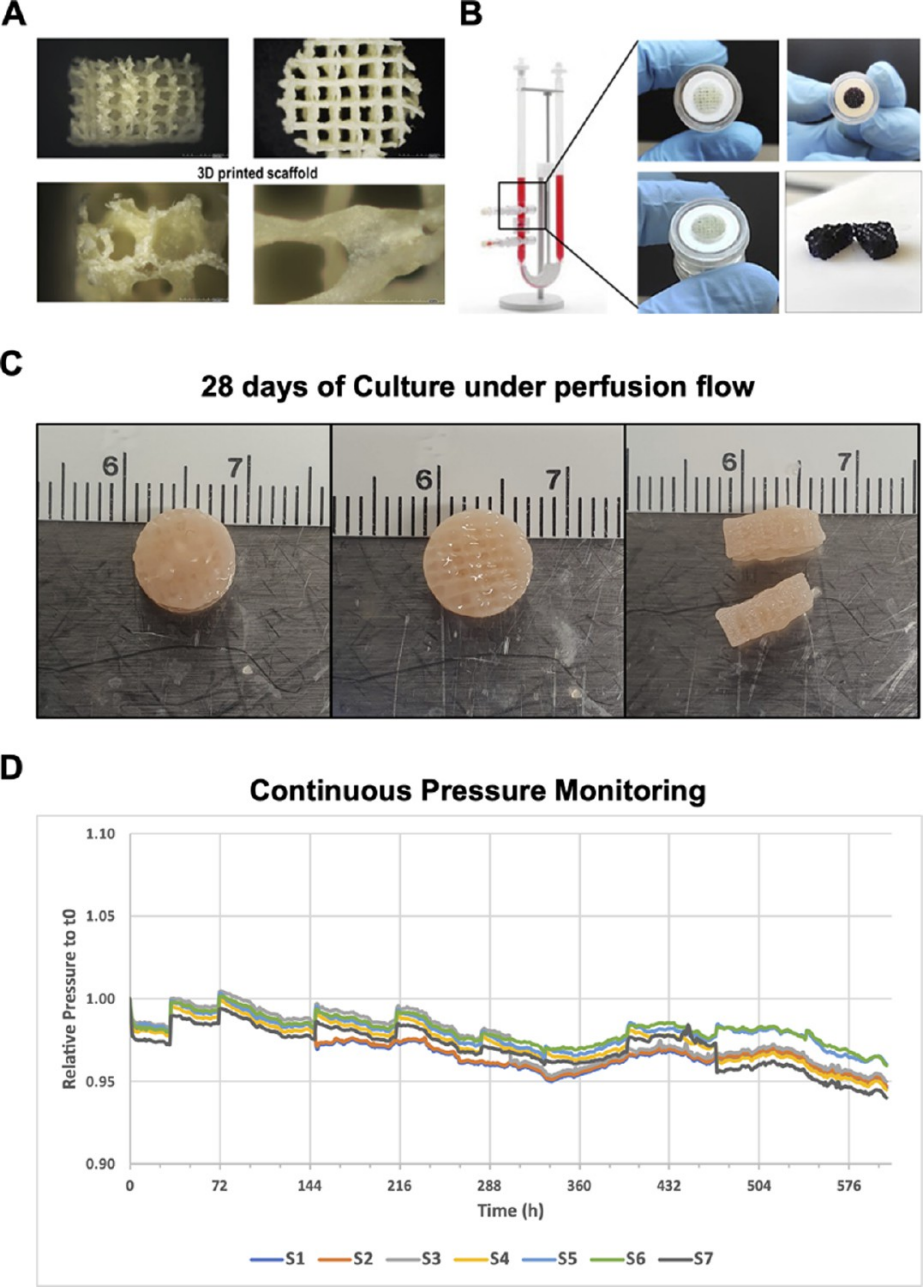
Integration and validation of 3D-printed bHAGel scaffolds in a dynamic perfusion bioreactor system. (A) Representative images of the 3D-printed bHAGel scaffold showing the macroporous architecture (top) and surface microtexture introduced via freeze-drying (bottom), both designed to support cell infiltration and perfusion; (B) the scaffold fits precisely within the perfusion chamber of the U-CUP bioreactor, enabling a sealed configuration. Blue dye staining demonstrates effective and homogeneous fluid distribution across the construct; (C) after 28 days of dynamic in vitro culture with MSOD MSCs, the scaffold retained its original cylindrical shape and appeared filled with tissue-like material, indicative of successful colonization and structural preservation; (D) relative pressure profiles (normalized to *t*_0_) recorded from seven independent bioreactor units (S1–S7) using a custom noninvasive pressure sensor. The stable traces indicate consistent perfusion across all scaffolds over the entire 28 day culture period. See also Supporting Information Figure S1 for an overview of the experimental workflow.

To evaluate the integration, structural robustness, and long-term perfusability of the 3D-printed bHAGel scaffold under dynamic culture conditions, we performed a 28 day perfusion experiment using the U-CUP bioreactor system. After 3D printing and freeze-drying, the bHAGel scaffold reveals a well-defined macroporous architecture and surface microtexture, both essential for cell infiltration and medium permeability (Figure 7A). The scaffold fits precisely within the culture chamber of the U-CUP bioreactor, ensuring a sealed configuration and enabling homogeneous perfusion, as demonstrated by a uniform blue dye distribution (Figure 7B). Following 28 days of dynamic in vitro culture with MSOD MSCs, the scaffold preserved its original geometry and exhibited tissue-mimetic properties (Figure 7C), suggesting robust mechanical stability and progressive cellular colonization throughout the construct.

We have monitored the relative pressure profiles acquired via a custom-built, noninvasive pressure sensor. All seven bioreactors (S1–S7) displayed stable traces over time, without major fluctuations or pressure buildups, indicating continuous scaffold perfusability and unimpeded flow across the 28-day culture period (Figure 7D).

These data confirm that the bHAGel scaffold maintains structural integrity and supports stable long-term perfusion under dynamic culture conditions. The consistency of the pressure profiles further validates the suitability of the construct for prolonged bioreactor-based tissue engineering applications.

### 3.3 Donor-Derived hMSCs Can be Cultured on the Biomimetic bHAGel Scaffold and Differentiated to Osteoblasts

To evaluate the potential of the bHAGel scaffold to support seeding and osteogenic differentiation, human BM-MSCs from three independent donors (BM187p3, BM231p3, and BM272p3) were cultured in a dynamic perfusion system for 21 days following a 5-day preconditioning phase (Figure 8). SEM imaging at day 0 revealed sparse cell adhesion and absence of the extracellular matrix (ECM), whereas by day 21, scaffolds were densely populated with cells and exhibited widespread ECM deposition. Surface morphology appeared mineral-like, with a fibrillar-to-granular texture consistent with early osteogenic maturation. Histological and ultrastructural analyses further supported these observations. H&E staining of constructs from all three donors (Figure S2) showed a clear progression from thin cellular linings with little ECM at Day 0 to dense eosinophilic matrix deposition bridging pores and surrounding scaffold struts at Day 21. Nuclei with a preserved morphology were homogeneously distributed within the newly formed matrix. Corresponding SEM images corroborated this trend, showing sparse cell adhesion and smooth scaffold surfaces at Day 0, whereas by Day 21, the surfaces were densely populated with interconnected fibrillar structures.

**Figure 8:**
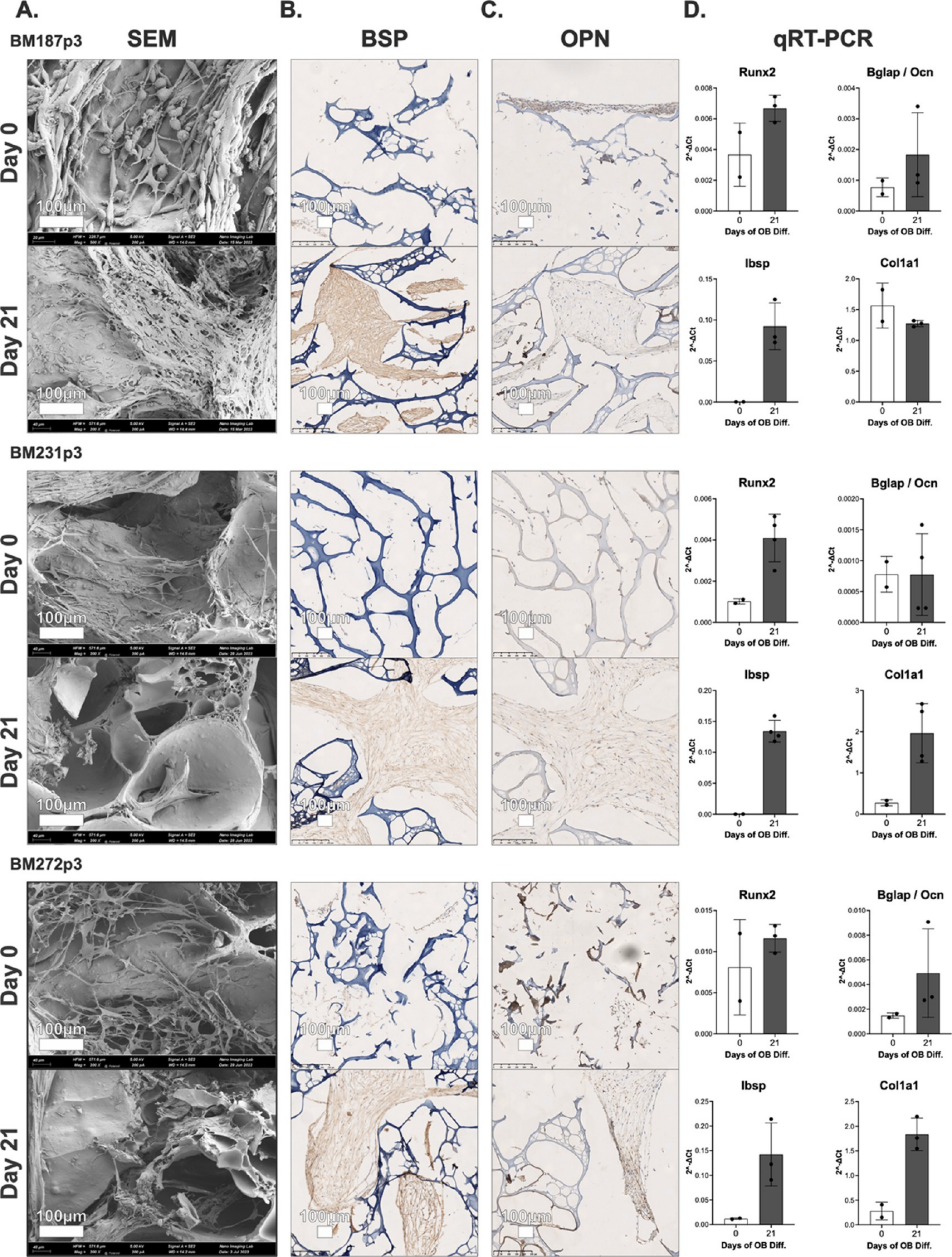
Multimodal analysis of osteogenic differentiation on 3D biomimetic bHAGel scaffolds cultured under dynamic perfusion. Column (A): Representative SEM images of scaffolds seeded with BM-MSCs from three donors (BM231p3, BM272p3, BM187p3), at day 0 and day 21. Constructs at day 21 show widespread cellular colonization and matrix deposition with surface features consistent with early mineralization. Column (B,C): Immunohistochemistry staining for bone sialoprotein (BSP) and osteopontin (OPN) at day 0 and day 21 for each donor. Minimal expression at day 0 contrasts with strong ECM-localized staining at day 21, indicating osteogenic progression. Column (D): qRT-PCR analysis of osteogenic markers Runx2, Ibsp, Bglap (osteocalcin), and Col1a1. Expression levels were normalized to GAPDH and calculated using the 2^–Δ*Ct*^ method. Consistent expression trends across donors indicate activation of osteogenic differentiation under dynamic conditions. Each plot represents data from one donor. Bars represent mean ± SD of biological replicates. Due to limited biological replicates (*n* = 2 for day 0; *n* = 3 for day 21), data are presented without statistical analysis.

Immunohistochemistry confirmed this transition: bone sialoprotein (BSP) and osteopontin (OPN) were undetectable at day 0 but strongly expressed at day 21, localized within ECM-rich regions. These findings were substantiated by qRT-PCR analysis, which demonstrated a consistent trend toward the upregulation of canonical osteogenic markers (Runx2, Ibsp, Col1a1, and Bglap) across all three donors. While statistical testing was not applied due to limited biological replicates (*n* = 2 at day 0; *n* = 3 at day 21), all donor-specific profiles followed a similar expression pattern, supporting the scaffold’s ability to induce osteogenic differentiation in a reproducible, donor-dependent manner.

Scaffold colonization, although substantial, remained incomplete, indicating that further optimization of the internal pore architecture may be required to enhance cell penetration and matrix distribution. These data confirm the compatibility of the bHAGel scaffold with a long-term dynamic culture and underscore the need to balance bioactivity and structural accessibility in future scaffold iterations.

### 3.4 bHAGel-Osteoblast Constructs Support Osteoclastogenesis under Physiological and Standard In Vitro Conditions

To assess whether the bHAGel-osteoblast construct could serve as a physiologically relevant in vitro model of bone remodeling, we first evaluated its ability to support the coexistence and differentiation of osteoclast-lineage cells under defined osteoclastogenic and osteotropic conditions. Human CD14^+^ monocytes were seeded onto scaffolds precultured with osteogenically differentiated MSCs and exposed to either osteoclastogenic (RM: M-CSF + RANKL) or osteotropic (VM: M-CSF + vitamin D3) conditions. The RM condition served as a positive control, representing the widely used in vitro system for direct osteoclastogenesis through exogenous RANKL,^58^ while the VM condition was designed to mimic a more physiological bone microenvironment, where Vitamin D3 acts on resident osteoblasts to induce RANKL expression and thereby stimulate osteoclast differentiation indirectly.^59^ Additional groups treated with RANKL or Vitamin D3 alone (R and V, respectively) in the absence of M-CSF were included as negative controls, allowing us to normalize the baseline effects of each soluble factor. Comparative qRT-PCR analysis was then performed on cells extracted from the scaffolds to assess expression of osteoclast and osteoblast-associated genes (Figure S3). This analysis revealed robust induction of TRAP in both VM and RM conditions relative to their controls (V and R), confirming that osteoclast differentiation occurred not only under direct stimulation but also via osteoblast-mediated pathways. Notably, OPN, associated with resorptive activity,^60^ was particularly elevated in VM conditions, suggesting enhanced physiological activation. CTSK was comparably expressed across groups with a slight reduction in RM.

In parallel, transcriptional profiling of osteoblastic markers (COL1A1, IBSP, BGLAP, and PTX3) revealed a divergence in osteoblastic phenotype between RM and VM scaffolds. While RM constructs expressed higher BGLAP, they showed downregulation of COL1A1 and IBSP and significantly elevated PTX3, potentially reflecting a more inflammatory or remodeling-prone OB state in response to supraphysiological RANKL.^61,62^ These findings confirm that the VM condition supports osteoclastogenesis via endogenous signaling,^63^ and that distinct osteoblast phenotypes emerge depending on the differentiation route, highlighting the biological relevance and versatility of this coculture system. Compared to other 3D-printed-scaffold-based approaches for modeling the osteoblastic and osteoclastic bone niche, our model demonstrates a more physiologically relevant (Vitamin-D3-mediated) pathway for differentiation of the osteoclastic population, also validating the function of osteoblasts and their precursors in the system along the RANK/RANKL/OPG pathway.^64–66^

The interplay between osteogenic and osteoclastic processes observed in this system is likely reinforced by both the bioactive composition of bHA and the dynamic environment used for the culture. Magnesium substitution within bHA has been shown to enhance alkaline phosphatase activity, collagen synthesis, and the expression of osteogenic markers while simultaneously modulating osteoclast precursor behavior through RANKL/OPG signaling and cytoskeletal organization, thereby influencing resorptive dynamics.^67^ In addition, convective nutrient/solute transport and interstitial shear stress generated during perfusion are known to promote osteogenic gene expression in mesenchymal progenitors and to regulate osteoclast activity.^68^ Altogether, these combined chemical and mechanical cues support a coordinated bone-like remodeling process within bHAGel scaffolds, bridging osteogenesis in a physiologically relevant manner.

### 3.5 Dynamic Coculture under Perfusion Confirms Osteoclast Differentiation and PTX3 Dynamics

Having established that osteoblast-mediated osteoclastogenesis can be achieved under static conditions in VM, we next validated the system under dynamic perfusion culture, incorporating multiple MSC donors and an extended analysis of osteoclastic function and inflammatory responses (Figure 9). Human CD14^+^ cells were seeded onto bHAGel scaffolds previously cultured for 26 days under osteogenic conditions and then maintained in osteotropic conditions for an additional 10 days (Figure 9A).

**Figure 9:**
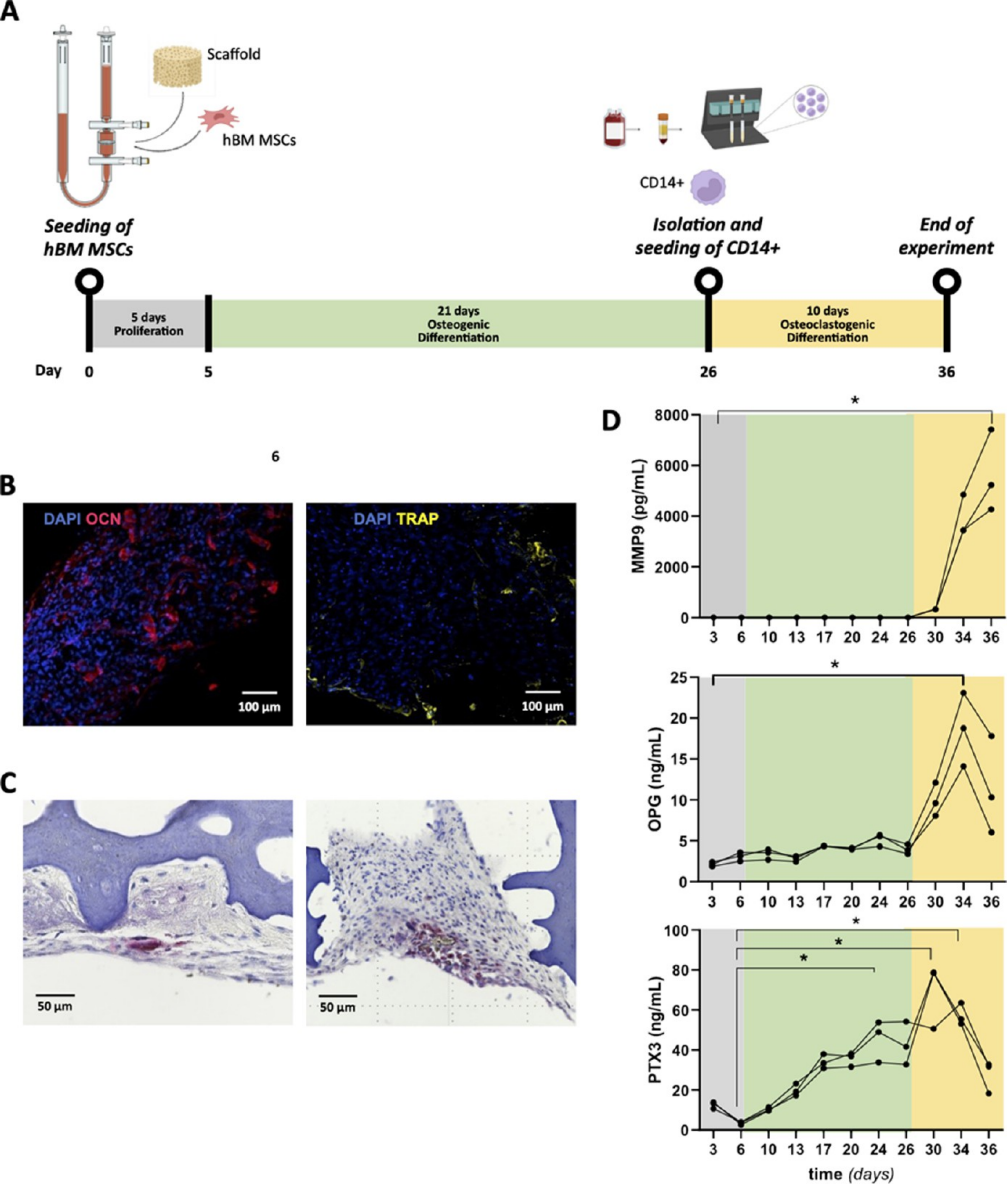
(A) Schematic representation of the experimental timeline for generating the osteoblast–osteoclast 3D coculture model; (B) representative whole-mount immunofluorescence (IF) staining of cell-seeded scaffolds with detection of markers of OBs (Osteocalcin, OCN, red) and OCs (TRAP, yellow) and DAPI-stained nuclei (blue); (C) representative TRAP5b-stained histological images of cell-seeded scaffolds harvested at the last time point. Positive cells are red-stained. (D) The concentration of selected bone turnover biomarkers (matrix metalloproteinase-9, MMP9, osteoprotegerin, OPG, and the long pentraxin 3, PTX3) in the 3D cell culture supernatant was measured at the indicated time points using the ELLA Automated Immunoassay System or a commercial ELISA kit (for PTX3). Data shown are from a single donor (BM272p3) and values in D are mean ± SEM, *n* = 2–3. *p <0.05, Kruskal–Wallis with Dunn’s multiple comparisons test.

Confocal IF on whole-mount constructs confirmed the coexistence of osteocalcin^+^ osteoblasts and TRAP^+^ osteoclasts (Figure 9B). Histochemical staining of TRAP5b on scaffold sections further corroborated the presence of enzymatically active osteoclasts (Figure 9C). To assess functional remodeling activity and inflammation, we quantified MMP9, OPG, and PTX3 levels in the supernatant over time (Figure 9D). As expected, MMP9 sharply increased between day 26 and 30, correlating with the onset of osteoclast differentiation. OPG levels also rose following monocyte seeding, consistent with osteoblast participation in OB–OC regulation.

Strikingly, PTX3 levels also increased during this window and then declined by day 36. PTX3 is upregulated during osteoblast differentiation and is also detectable, albeit at significantly lower levels (approximately 6- to 10-fold lower), in maturing osteoclasts.^62^ This expression profile is therefore consistent with previously reported kinetics. Immunostaining further confirmed localization of PTX3 within and around DAPI^+^ cells in cell-seeded constructs, but not in acellular scaffolds (Figure S4), supporting its cell-derived origin and incorporation into a nascent extracellular matrix. While other in vitro models have been used to study the role of PTX3 in the bone niche, to our knowledge, our approach is the first one to examine the complex dynamics of PTX3 signaling in a 3D coculture of osteoblasts and osteoclasts.^69,70^

## 4 Conclusions

Altogether, our work presents a standardized biomimetic injectable material designed for compatibility with 3D printing and perfused bioreactors, which are suitable for long-term cultures and osteogenic support. These features position bHAGel matrices as modular tools for the fabrication of personalized in vitro bone-like systems with potential applications in tissue engineering, disease modeling, and drug testing.

Our results demonstrate that bHAGel 3D-printed scaffolds provide a robust and versatile platform for engineering bone-mimetic constructs under dynamic culture. By integrating biomimetic chemistry, printability, and long-term perfusability, this system provides a scalable solution for modeling osteogenesis and bone remodeling in vitro. The gelatin component acts as a biodegradable, cell-adhesive matrix that promotes mesenchymal stem cell attachment and differentiation, while bHA particles contribute as rheological modulation and osteoinductive cues. Combined with the structural hierarchy introduced by 3D printing and freeze-drying, this provides a suitable microenvironment for both cellular organization and functional differentiation. Future developments will harness the high print fidelity and tunable rheology of bHAGel to fabricate next-generation bioarchitectures with increasing spatial and functional complexity. This includes multiscale scaffolds with gradient porosity, compartmentalized niches, and embedded vasculature-mimetic channels tailored for spatially resolved cell seeding and localized biochemical cues. By incorporating tool-path-level modulation and multiphase printing, the platform could support zonal tissue engineering (e.g., osteochondral or osteoimmune interfaces), enabling the design of truly biomimetic organoid environments. These advances will expand the utility of bHAGel matrices from bone regeneration to the controlled modeling of pathophysiological bone conditions and beyond.

## Supplementary Material



## Data Availability

The data that support the findings of this study are available from the corresponding author upon reasonable request.

## Notes

SEM images of bHA hybrid particles focusing on the macro view to the particles’ surface; histological and ultrastructural evidence of sustained viability and matrix deposition in perfused constructs; gene expression analysis of bone cells cultured in bHAGel scaffolds in osteoblastic and osteoclastic differentiation medium cultured in static condition; and representative fluorescence microscopy images cell-seeded and of empty scaffold sections (PDF)
