# Supplementary material for: Injectable Nanocomposite Biomaterial for 3D Printing of Personalized Matrices and Their Use in Bioreactors for Bioengineering Advanced Cell Culture Models
Source: ACS Appl Mater Interfaces. 2025 Dec 8;17(50):68440–56. doi: 10.1021/acsami.5c18437 (PMC12723638; doi:10.1021/acsami.5c18437)
Supplement: Supplementary file 1 [file am5c18437_si_001.pdf]

Supporting Information

Injectable nanocomposite biomaterial for 3D printing of personalized matrices and their use in bioreactors for bioengineering advanced cell culture models

Elisabetta Campodoni[a†], Andrea Mazzoleni[b†], Margherita Montanari[a], Gaia Vicinelli[a], Valentina Possetti[c], Antonio Inforzato[c], Ivan Martin[b,d], Manuele G. Muraro[d], Monica Sandri[a*]*

[a] Institute of Science, Technology and Sustainability for Ceramics (ISSMC) – National Research Council (CNR), Faenza (RA), Italy

[b] Department of Biomedical Engineering, University of Basel, Basel, Switzerland

[c] Department of Biomedical Sciences, Humanitas University, 20072 Pieve Emanuele, Italy IRCCS Humanitas Research Hospital, 20089, Rozzano (MI), Italy

[d] Tissue Engineering, Department of Biomedicine, University of Basel and University Hospital Basel, Basel, Switzerland

[†] E.C and A.M contributed equally to this paper.

Corresponding authors:
Elisabetta Campodoni and Monica Sandri,
Institute of Science, Technology and Sustainability for Ceramics (ISSMC) – National Research Council (CNR), Faenza (RA), Italy
elisabetta.campodoni@issmc.cnr.it monica.sandri@issmc.cnr.it

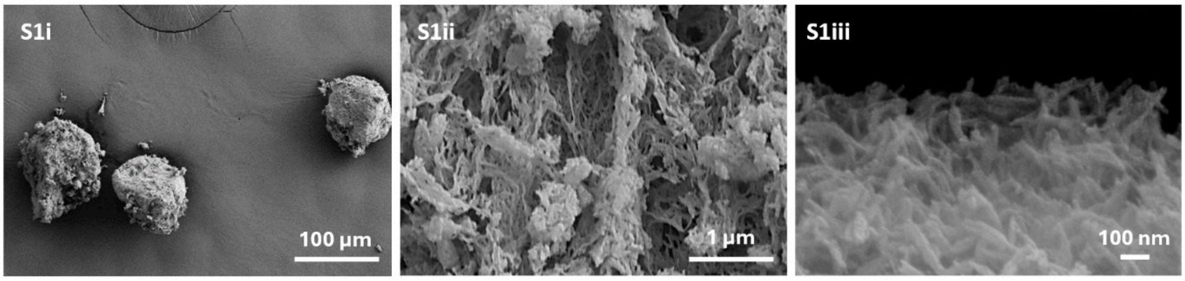

Figure S1: SEM images of bHA hybrid particles at different magnifications: S1i shows a macro view of the hybrid microparticles; S1ii highlights the presence of gelatin; S1iii shows a higher magnification of the nanostructures on the particles' surface.

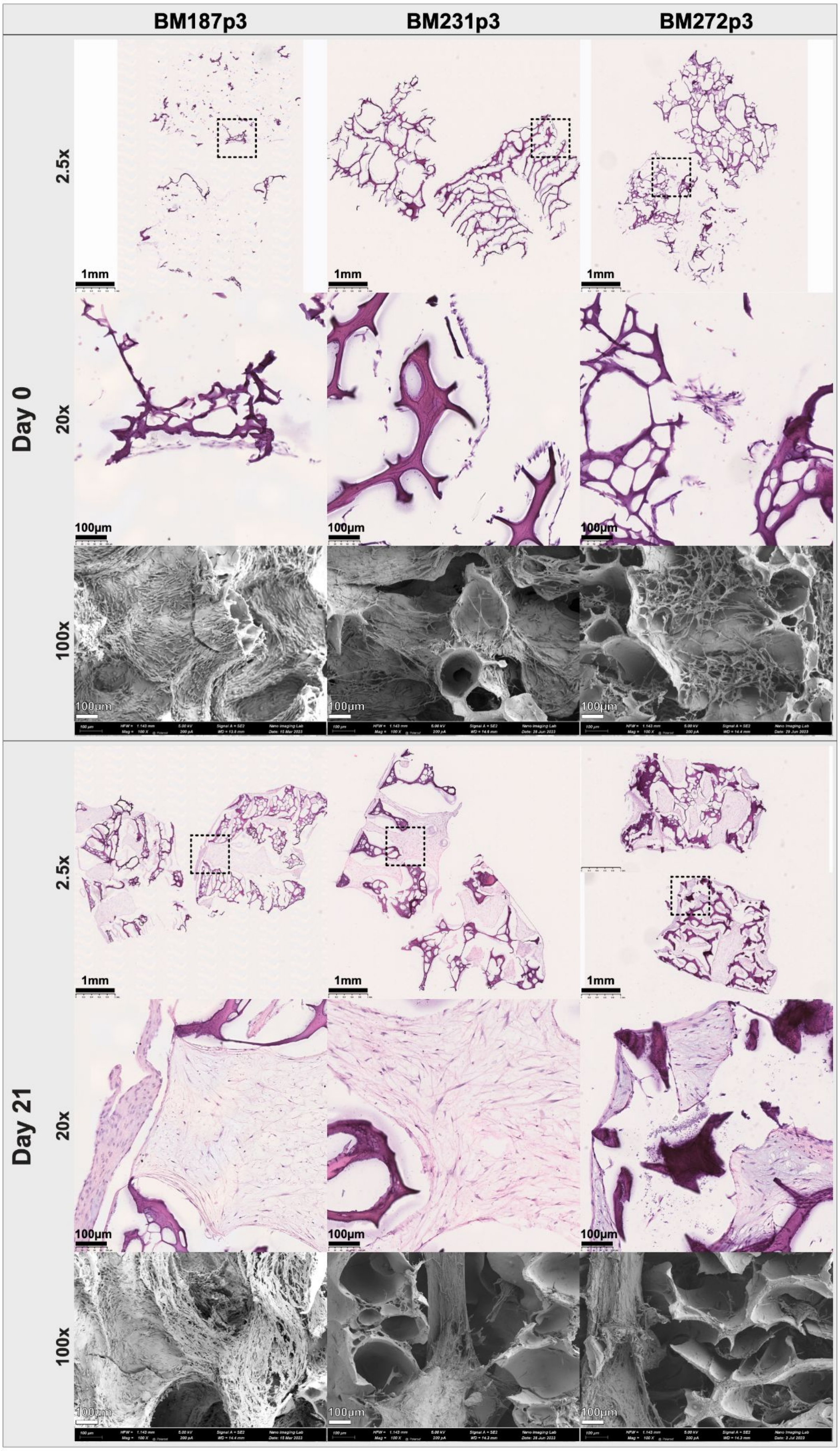
BM187p3
BM231p3
BM272p3
Day 0
Day 21
2.5x
20x
100x
1mm
100μm

Figure S2. Histological and ultrastructural evidence of sustained viability and matrix deposition in perfused constructs. Representative hematoxylin–eosin (H&E) and scanning electron microscopy (SEM) images of cell-laden hydrogel–ceramic scaffolds from three independent bone-marrow donors (BM187, BM231, BM272) at Day 0 (end of proliferation phase) and Day 21 (after osteogenic differentiation under perfusion). Low-magnification (2.5x) views show overall scaffold colonization, and corresponding high-magnification (20x) insets reveal tissue organization. SEM images (100x) illustrate surface morphology and extracellular matrix (ECM) architecture at matching time points. At Day 0, constructs display sparse cellular coverage, smooth scaffold surfaces, and minimal ECM deposition. By Day 21, all donors exhibit dense eosinophilic ECM bridging pores, abundant nuclei with normal morphology, and extensive fibrillar structures on scaffold surfaces, indicating sustained cell activity and maintained perfusion pathways. Scale bars: 1 mm (2.5x); 100 µm (20x, 100x).

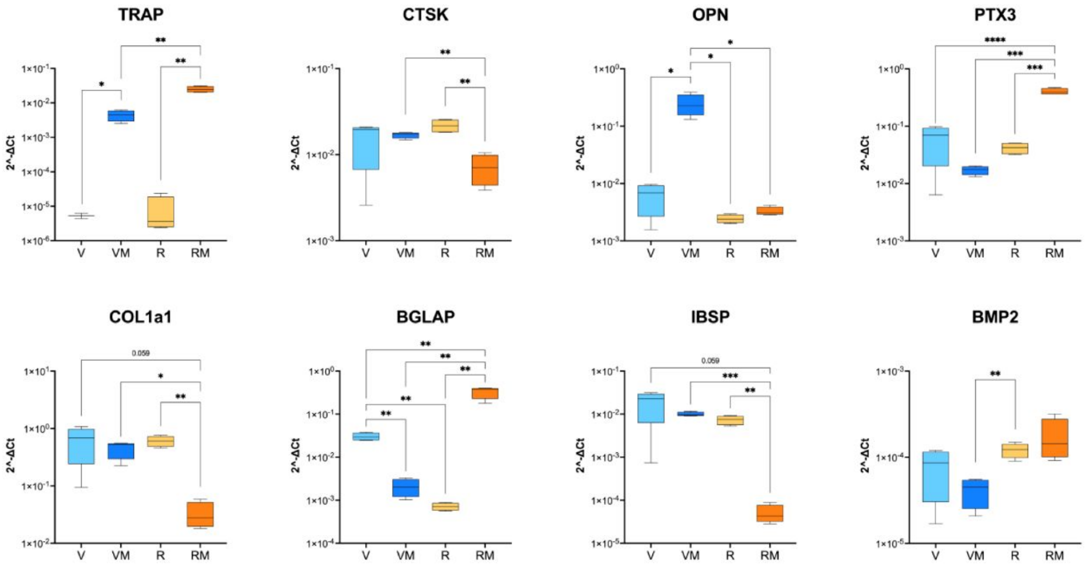

Figure S3. Gene expression analysis of bone cells cultured in bHAGel scaffolds in osteoblastic and osteoclastic differentiation medium cultured in static conditions. qRT-PCR analysis of osteoclastic markers *Trap*, *Ctsk*, *Opn*, inflammatory marker *Ptx3,* and osteoblastic markers *Col1a1, Bglap, Ibsp,* and *Bmp2*. Expression levels were normalized to *GAPDH* and calculated using the 2^–ΔCt method. Each plot represents data from one donor. Bars represent mean ± SD of biological replicates (n=4). Statistical analysis was done via Welch's t-test, with statistically significant differences highlighted in the graphs (*: $p < 0.05$; **: $p < 0.005$, ***: $p < 0.0005$).

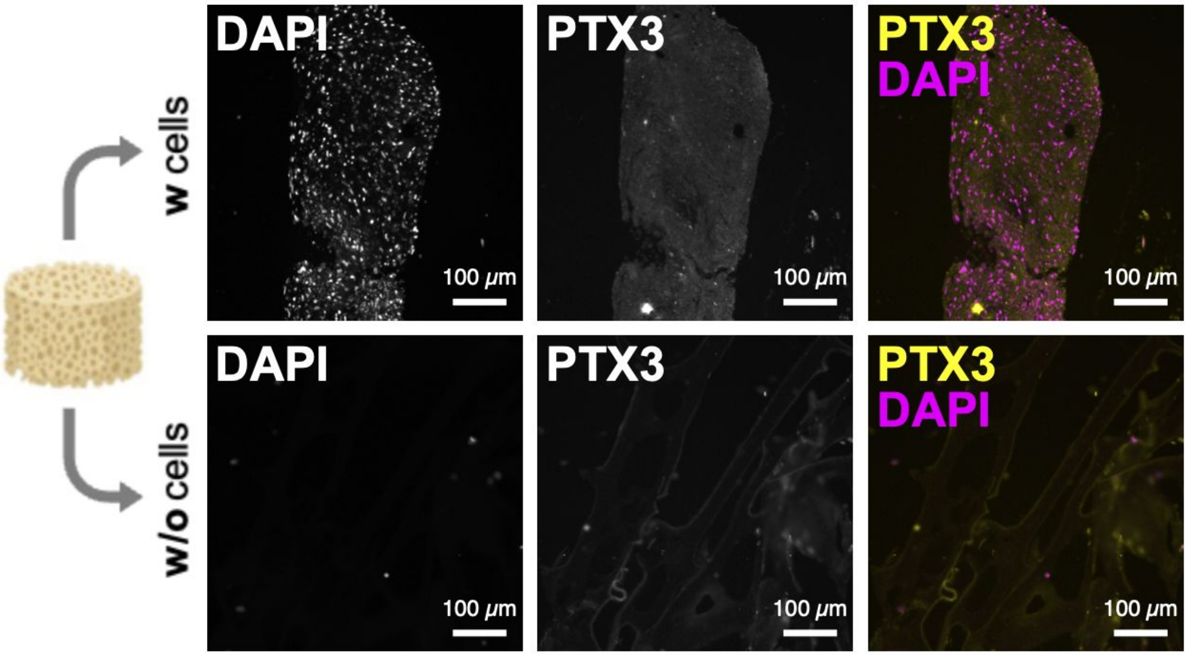

Figure S4. Representative fluorescence microscopy images cell-seeded and of empty scaffold sections showing extra- and peri-cellular localization of PTX3 staining. Scale bar: 100 μm.
